# Impact of COVID-19 in patients on active melanoma therapy and with history of melanoma

**DOI:** 10.1186/s12885-023-10708-6

**Published:** 2023-03-23

**Authors:** Douglas B. Johnson, Michael B. Atkins, Cassandra Hennessy, Trisha Wise-Draper, Hannah Heilman, Joy Awosika, Ziad Bakouny, Chris Labaki, Renee Maria Saliby, Clara Hwang, Sunny R. K. Singh, Nino Balanchivadze, Christopher R. Friese, Leslie A. Fecher, James J. Yoon, Brandon Hayes-Lattin, Mehmet A. Bilen, Cecilia A. Castellano, Gary H. Lyman, Lisa Tachiki, Sumit A. Shah, Michael J. Glover, Daniel B. Flora, Elizabeth Wulff-Burchfield, Anup Kasi, Saqib H. Abbasi, Dimitrios Farmakiotis, Kendra Viera, Elizabeth J. Klein, Lisa B. Weissman, Chinmay Jani, Matthew Puc, Catherine C. Fahey, Daniel Y. Reuben, Sanjay Mishra, Alicia Beeghly-Fadiel, Benjamin French, Jeremy L. Warner, COVID-19 and Cancer Consortium, Sonya A. Reid, Alaina J. Brown, Alex Cheng, Sarah Croessmann, Elizabeth J. Davis, Kyle T. Enriquez, Erin A. Gillaspie, Daniel Hausrath, Xuanyi Li, David A. Slosky, Carmen C. Solorzano, Matthew D. Tucker, Karen Vega-Luna, Lucy L. Wang, Trisha M. Wise-Draper, Syed A. Ahmad, Punita Grover, Shuchi Gulati, Jordan Kharofa, Tahir Latif, Michelle Marcum, Davendra P. S. Sohal, Olga Zamulko, Toni K. Choueiri, Jean M. Connors, George D. Demetri, Narjust Duma, Dory A. Freeman, Antonio Giordano, Alicia K. Morgans, Anju Nohria, Renee-Maria Saliby, Andrew L. Schmidt, Eliezer M. Van Allen, Wenxin Xu, Rebecca L. Zon, Shirish M. Gadgeel, Sheela Tejwani, Anne Boldt, Aaron M. Cohen, Shannon McWeeney, Eneida R. Nemecek, Staci P. Williamson, Deepak Ravindranathan, Jerome J. Graber, Petros Grivas, Jessica E. Hawley, Elizabeth T. Loggers, Ryan C. Lynch, Elizabeth S. Nakasone, Michael T. Schweizer, Shaveta Vinayak, Michael J. Wagner, Albert Yeh, Elwyn C. Cabebe, Michael J. Glover, Alokkumar Jha, Ali Raza Khaki, Lidia Schapira, Julie Tsu-Yu Wu, Goetz Kloecker, Barbara B. Logan, Chaitanya Mandapakala, Crosby D. Rock, Panos Arvanitis, Pamela C. Egan, Hina Khan, Adam J. Olszewski, Kendra Vieira, Lisa B. Weissmann, Padmanabh S. Bhatt, Melissa G. Mariano, Carey C. Thomson, Theresa M. Carducci, Karen J. Goldsmith, Susan Van Loon, Mariam Alexander, Sara Matar, Sarah Mushtaq, Keith E. Stockerl-Goldstein, Omar Butt, Mark A. Fiala, Jeffrey P. Henderson, Ryan S. Monahan, Alice Y. Zhou, Philip E. Lammers, Sanjay G. Revankar, Salvatore A. Del Prete, Michael H. Bar, Anthony P. Gulati, K. M. Steve Lo, Suzanne J. Rose, Jamie Stratton, Paul L. Weinstein, Shilpa Gupta, Nathan A. Pennell, Manmeet S. Ahluwalia, Scott J. Dawsey, Christopher A. Lemmon, Amanda Nizam, Nima Sharifi, Claire Hoppenot, Ang Li, Susan Halabi, Hannah Dzimitrowicz, Tian Zhang, Sharad Goyal, Minh-Phuong Huynh-Le, Peter Paul Yu, Jessica M. Clement, Ahmad Daher, Mark E. Dailey, Rawad Elias, Asha Jayaraj, Emily Hsu, Alvaro G. Menendez, Oscar K. Serrano, Melissa K. Accordino, Divaya Bhutani, Dawn Hershman, Matthew A. Ingham, Gary K. Schwartz, Eric H. Bernicker, John F. Deeken, Danielle Shafer, Erika Ruíz-García, Ana Ramirez, Diana Vilar-Compte, Mark A. Lewis, Terence D. Rhodes, David M. Gill, Clarke A. Low, Sandeep H. Mashru, Abdul-Hai Mansoor, Grant C. Lewis, Stephanie J. Smith, Howard A. Zaren, Gayathri Nagaraj, Mojtaba Akhtari, Dan R. Castillo, Eric Lau, Mark E. Reeves, Stephanie Berg, Natalie Knox, Timothy E. O’Connor, Eric B. Durbin, Amit A. Kulkarni, Heather H. Nelson, Zohar Sachs, Rachel P. Rosovsky, Kerry L. Reynolds, Aditya Bardia, Genevieve Boland, Justin F. Gainor, Leyre Zubiri, Thorvardur R. Halfdanarson, Tanios S. Bekaii-Saab, Aakash Desai, Irbaz B. Riaz, Surbhi Shah, Katherine E. Smith, Colt Williams, Nathaniel Bouganim, Arielle Elkrief, Justin Panasci, Donald C. Vinh, Gregory J. Riely, Rimma Belenkaya, John Philip, Bryan Faller, Rana R. McKay, Archana Ajmera, Sharon S. Brouha, Sharon Choi, Albert Hsiao, Seth Kligerman, Taylor K. Nonato, Erin G. Reid Sibel Blau, Sachin R. Jhawar, Daniel Addison, James L. Chen, Margaret E. Gatti-Mays, Vidhya Karivedu, Vidhya Karivedu, Joshua D. Palmer, Daniel G. Stover, Sarah Wall, Nicole O. Williams, Monika Joshi, Hyma V. Polimera, Lauren D. Pomerantz, Marc A. Rovito, Elizabeth A. Griffiths, Pragati G. Advani, Igor Puzanov, Salma K. Jabbour, Christian F. Misdary, Mansi R. Shah, Gerald Batist, Erin Cook, Miriam Santos Dutra, Cristiano Ferrario, Wilson H. Miller, Babar Bashir, Christopher McNair, Sana Z. Mahmood, Vasil Mico, Andrea Verghese Rivera, Natasha C. Edwin, Melissa Smits, Deborah B. Doroshow, Matthew D. Galsky, Michael Wotman, Alyson Fazio, Julie C. Fu, Kathryn E. Huber, Mark H. Sueyoshi, Vadim S. Koshkin, Hala T. Borno, Daniel H. Kwon, Eric J. Small, Sylvia Zhang, Samuel M. Rubinstein, William A. Wood, Tessa M. Andermann, Christopher Jensen, Daniel W. Bowles, Christoper L. Geiger, Lawrence E. Feldman, Kent F. Hoskins, Gerald Gantt, Li C. Liu, Mahir Khan, Ryan H. Nguyen, Mary Pasquinelli, Candice Schwartz, Neeta K. Venepalli, Blanche H. Mavromatis, Ragneel R. Bijjula, Qamar U. Zaman, David M. Aboulafiam, Brett A. Schroeder, Umit Topaloglu, Saif I. Alimohamed, Joan K. Moore, Prakash Peddi, Lane R. Rosen, Briana Barrow McCollough, Navid Hafez, Roy Herbst, Patricia LoRusso, Maryam B. Lustberg, Tyler Masters, Catherine Stratton

**Affiliations:** 1 Vanderbilt University Medical Center, Nashville, TN 37232 USA; 2 Georgetown Lombardi Comprehensive Cancer Center, Georgetown University, Washington, DC USA; 3 University of Cincinnati Cancer Center, Cincinnati, USA; 4 Dana-Farber Cancer Institute, Boston, MA USA; 5 Henry Ford Cancer Institute, Henry Ford Hospital, Detroit, MI USA; 6 University of Michigan Rogel Cancer Center, Ann Arbor, MI USA; 7 Knight Cancer Institute, Oregon Health and Science University, Portland, OR USA; 8 Winship Cancer Institute, Emory University, Atlanta, GA USA; 9 Fred Hutchinson Cancer Center, Seattle, WA USA; 10 Stanford Cancer Institute, Stanford University, Stanford, CA USA; 11 St. Elizabeth Healthcare, Edgewood, KY USA; 12 The University of Kansas Cancer Center, Lawrence, KS USA; 13 Brown University and Lifespan Cancer Institute, Providence, Rhode Island USA; 14 Mount Auburn Hospital, Cambridge, MA USA; 15 Virtua Health, Marlton, NJ USA; 16 Hollings Cancer Center, Medical University of South Carolina, Charleston, SC USA

**Keywords:** COVID-19, Melanoma, Immune therapy, Targeted therapy, Cancer

## Abstract

**Introduction:**

COVID-19 particularly impacted patients with co-morbid conditions, including cancer. Patients with melanoma have not been specifically studied in large numbers. Here, we sought to identify factors that associated with COVID-19 severity among patients with melanoma, particularly assessing outcomes of patients on active targeted or immune therapy.

**Methods:**

Using the COVID-19 and Cancer Consortium (CCC19) registry, we identified 307 patients with melanoma diagnosed with COVID-19. We used multivariable models to assess demographic, cancer-related, and treatment-related factors associated with COVID-19 severity on a 6-level ordinal severity scale. We assessed whether treatment was associated with increased cardiac or pulmonary dysfunction among hospitalized patients and assessed mortality among patients with a history of melanoma compared with other cancer survivors.

**Results:**

Of 307 patients, 52 received immunotherapy (17%), and 32 targeted therapy (10%) in the previous 3 months. Using multivariable analyses, these treatments were not associated with COVID-19 severity (immunotherapy OR 0.51, 95% CI 0.19 – 1.39; targeted therapy OR 1.89, 95% CI 0.64 – 5.55). Among hospitalized patients, no signals of increased cardiac or pulmonary organ dysfunction, as measured by troponin, brain natriuretic peptide, and oxygenation were noted. Patients with a history of melanoma had similar 90-day mortality compared with other cancer survivors (OR 1.21, 95% CI 0.62 – 2.35).

**Conclusions:**

Melanoma therapies did not appear to be associated with increased severity of COVID-19 or worsening organ dysfunction. Patients with history of melanoma had similar 90-day survival following COVID-19 compared with other cancer survivors.

**Supplementary Information:**

The online version contains supplementary material available at 10.1186/s12885-023-10708-6.

## Introduction

SARS-CoV-2, the virus responsible for the COVID-19 pandemic, has resulted in particularly severe morbidity and mortality for patients at increased age, and with health co-morbidities, such as obesity, diabetes, cardiovascular disease including cardiomyopathy, and cancer [1]. A growing number of large studies have assessed the impact of COVID-19 among patients with various types of cancer, and among patients with various modalities of anti-cancer therapy [2, 3]. Given the diverse types of malignancy and treatment scenarios, many questions remain, particularly in less common tumor types.

Melanoma is the most lethal type of skin cancer and is an archetypal malignancy for the development of both targeted therapy (with BRAF and MEK inhibitors) and especially immunotherapy. Melanoma was the first malignancy where many checkpoint inhibitors were first approved and most aggressively combined (including agents targeting programmed death-1 [PD-1], cytotoxic T lymphocyte antigen-4 [CTLA-4], and lymphocyte antigen gene-3 [LAG-3]). In addition, melanoma have unique risk factors that differ from those for other malignancies (e.g., exposure to ultraviolet light rather than cigarette smoke, alcohol, obesity, or chronic inflammation), thus theoretically conferring a better prognosis.

Leveraging detailed patient reports to the COVID-19 and Cancer Consortium (CCC-19) registry—consisting of 12,661 patients with cancer and COVID-19 reported from 93 different centers, including 307 patients with melanoma—we sought to assess the factors, including recent targeted or immunotherapy, that were associated with COVID-19 severity and survival. We also assessed whether these therapies synergized with COVID-19 to produce exacerbated organ dysfunction. Finally, we compared survival following COVID-19 among patients with melanoma to those with all other cancer types in the database.

## Methods

### Study design

This was a registry-based retrospective cohort study that used data from the CCC19 registry, a centralized multi-institutional registry of patients who had COVID-19 and a diagnosis of cancer (either past or current). The registry is maintained as an electronic database using REDCap Software at Vanderbilt University Medical Center, and its schema and format have been previously described [4]. Records for the present study were accrued from March 17, 2020, to December 31, 2021, and included patients with a diagnosis of SARS-CoV-2 infection confirmed by serology or polymerase chain reaction tests. Patients with non-invasive cancers including non-melanoma skin cancer, in situ carcinoma, or precursor hematologic neoplasms were excluded. The analysis also excluded patients with inadequate data quality (quality score ≥ 5 according to our previously defined metric [4]) and those with incomplete follow-up to assess the outcomes of interest. Patients with cancer types other than melanoma were excluded when assessing the associations between modality of systemic anti-cancer therapy with COVID-19 severity among patients with melanoma. Patients with active cancer status or active systemic anti-cancer therapy were excluded when assessing the association between presence of non-active melanoma verses non-active other cancers with 30-day and 90-day mortality.

This study was approved by Institutional Review Board of Vanderbilt University Medical Center and participating sites. Informed consent was waived since data were anonymized and posed minimal risk to study participants.

### Data elements

The primary outcome was a 6-level ordinal scale of COVID-19 severity based on a patient’s most severe reported disease status: none of the complications listed here, hospital admission without supplemental oxygen, hospital admission with supplemental oxygen, intensive care unit admission, mechanical ventilation use, and death from any cause. Secondary outcomes were death from any cause within 30 days and 90 days after COVID-19 diagnosis. Class of recent (i.e., within 3 months prior to COVID-19 diagnosis) systemic anti-cancer therapy was defined as cytotoxic chemotherapy, targeted therapy, immunotherapy, or none [3].

### Statistical analysis

All statistical analysis methods and data elements were pre-specified in a statistical analysis plan. Standard descriptive statistics were used to summarize baseline characteristics and outcomes among patients with melanoma: categorical and continuous variables were summarized as counts (%) and median (interquartile range [IQR]), respectively. Laboratory measurements (BNP, CRP, troponin) and receipt of supplemental oxygen were summarized among patients with melanoma hospitalized at baseline. Ordinal logistic regression models with an offset for (log) follow-up time were used to determine whether class of recent anti-cancer therapy was associated with COVID-19 severity [5]. The model was adjusted for melanoma as the primary cancer type, age (quadratic term to accommodate a non-linear association), sex, race and ethnicity, geographical region of patient residence (defined as Sunbelt region or not), smoking status, obesity, comorbidities (cardiovascular, pulmonary. renal disease, diabetes, immunosuppression), Eastern Cooperative Oncology Group (ECOG) performance status, cancer status (remission or no evidence of disease, active and stable/responding, active and progressing, or unknown), and time period of COVID-19 diagnosis. Binary logistic regression models were used to determine whether type of non-active cancer (melanoma or other) was associated with 30-day and 90-day mortality. In addition to the covariates listed above but not including cancer status, the model was adjusted for corticosteroids as a COVID-19 treatment ever. Variables were assessed for collinearity before inclusion in multivariable models. Multiple imputation (10 imputations, missingness rates < 6%) using additive regression, bootstrapping, and predictive mean matching was used to impute missing and unknown data, except unknown ECOG status and unknown cancer status, which were included as “unknown” categories. Results were combined using Rubin’s rules and are reported as odds ratios (ORs) with 95% confidence intervals (CIs). All tests were two-sided and a 95% CI that did not cross 1.0 was considered significantly significant. All analyses were conducted using R version 4.0.2 (R Foundation for Statistical Computing, Vienna, Austria), including the Hmisc and rms extension packages.

## Results

We identified 307 patients with melanoma in the CCC19 registry. Among these, median age was 64 years (IQR 55–75 years), 167 (54%) were male, 14% were races other than non-Hispanic White, and 81 (26%) resided in the US Sunbelt region, Spain, or Mexico whereas 225 (73%) were from US states outside the sunbelt or Canada (Table 1). Regarding comorbid conditions, 105 (34%) had obesity, 69 (22%) had cardiovascular conditions, 56 (18%) had pulmonary comorbidities, and 56 (18%) had diabetes. In terms of melanoma disease status, 168 (55%) were in remission or had no evidence of disease, 70 (23%) had active melanoma that was stable or responding, and 37 (12%) had active melanoma that was progressing. Regarding melanoma treatment, 86 (28%) had systemic therapy in the 3 months preceding COVID-19 diagnosis, including immunotherapy (*n* = 52, 17%), targeted therapy (*n* = 32, 10%), or cytotoxic chemotherapy (*n* = 11, 4%).

**Table 1:** Demographics for all melanoma patients

Characteristics	All patients
	*N* = 307
Age [Median (IQR)]	64 (55–75)
Sex	
Female	140 (46%)
Male	167 (54%)
Race	
Non-Hispanic White	259 (84%)
Non-Hispanic Black	8 (3%)
Hispanic	14 (5%)
Other	22 (7%)
Missing/Unknown	4 (1%)
Geographic location	
US Sunbelt region, Spain, or Mexico	81 (26%)
US states outside of Sunbelt region or Canada	225 (73%)
Missing/Unknown	1 (0%)
Smoking status	
Never	179 (58%)
Current or Former	118 (38%)
Missing/Unknown	10 (3%)
Obesity	
Not obese	200 (65%)
Obese	105 (34%)
Missing/Unknown	2 (1%)
Comorbidities	
Diabetes mellitus	56 (18%)
Pulmonary comorbidities	56 (18%)
Cardiovascular comorbidities	69 (22%)
Renal comorbidities	38 (12%)
Immunosuppressed	44 (14%)
Missing/Unknown	2 (1%)
ECOG Status	
0	129 (42%)
1	62 (20%)
2 +	28 (9%)
Unknown	87 (28%)
Missing	1 (0%)
Cancer Status	
Remission/NED	168 (55%)
Active, stable/responding	70 (23%)
Active, progressing	37 (12%)
Unknown	32 (10%)
Recent systemic cancer therapy	
Systemic therapy in the last 3 months	86 (28%)
Cytotoxic Therapy	11 (4%)
Targeted Therapy	32 (10%)
Immunotherapy	52 (17%)
No systemic therapy in the last 3 months	215 (70%)
Missing/Unknown	6 (2%)
COVID-19 treatments	
Low-dose steroids ever	
Yes	41 (13%)
No	256 (83%)
Missing/Unknown	10 (3%)
High-dose steroids ever	
Yes	37 (12%)
No	254 (83%)
Missing/Unknown	16 (5%)
Monoclonal antibodies	
Yes	10 (3%)
No	290 (94%)
Missing/Unknown	7 (2%)
Timing of COVID diagnosis	
Jan—Apr 2020	63 (21%)
May—Aug 2020	102 (33%)
Sep—Dec 2020	86 (28%)
Jan—Apr 2021	32 (10%)
May—Aug 2021	15 (5%)
Sep—Dec 2021	9 (3%)

**Fig. 1:**
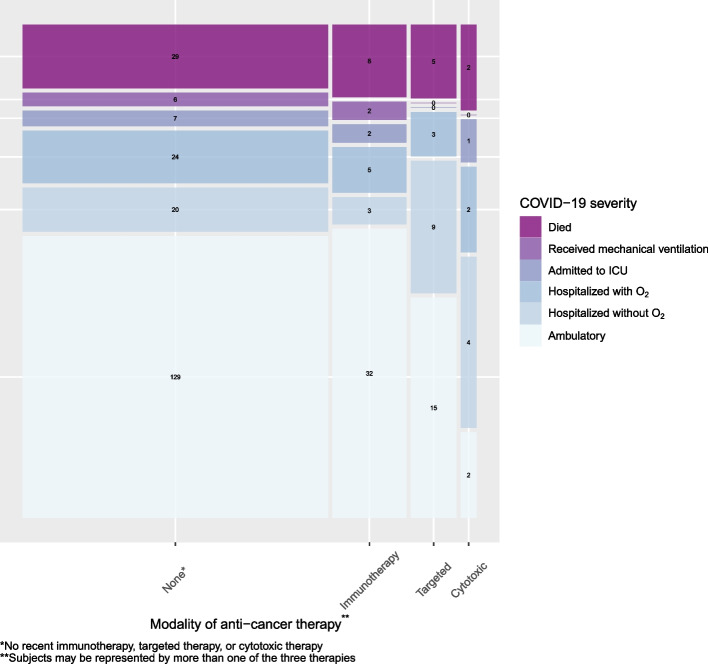
Distribution of the ordinal COVID-19 severity outcome, stratified by modality of anti-cancer therapy received within 3 months prior to COVID-19 diagnosis. Note: The ordinal COVID-19 severity outcome was based on a patient’s most severe reported disease status during follow-up (for example, a patient who was hospitalized and later died is included in “Died”). The width of the boxes is proportional to the number of patients who received each anti-cancer therapy; the height of the boxes is proportional to the number of patients in each outcome level. Patient numbers are listed for each outcome level

Among the 307 patients with melanoma, over a median follow up of 90 days from COVID-19 diagnosis, 127 (41%) were hospitalized at any time, including 85 (28%) that required supplemental oxygen, 40 (13%) that required ICU admission, and 44 (14%) that died due to any cause (Table S1). Among hospitalized patients, attribution was noted as definitely related to COVID-19 (*n* = 91, 72%), possibly related to COVID-19 (*n* = 26, 20%), and unrelated to COVID-19 (*n* = 10, 8%). COVID-19 severity is summarized by class of recent anti-cancer therapy in Fig. 1. On multivariable analyses, no class of therapy was associated with COVID-19 severity, including for immunotherapy (OR 0.51, 95% CI 0.19 – 1.39), targeted therapy (OR 1.89, 95% CI 0.64 – 5.55), or chemotherapy (OR 0.63, 95% CI 0.18 – 2.19) (Table 2). Age exhibited a significant non-linear (i.e., linear-quadratic) association with COVID-19 severity, such that older patients had higher COVID-19 severity. Pulmonary comorbidities (OR 2.27, 95% CI 1.12 – 4.62), diabetes (OR 2.12, 95% CI 1.01 – 4.47), ECOG 2 + (vs. ECOG 0; OR 12.5, 95% CI 4.22 – 37.2), active and progressing cancer (vs. remission, OR 3.22, 95% CI 1.21 – 8.58) were all associated with higher COVID-19 severity, whereas location in the sunbelt region (OR 0.35, 95% 0.17 – 0.75) and later diagnosis (after May 2020) was associated with lower COVID-19 severity (OR 0.10 – 0.18 compared with Jan – April 2020).

**Table 2:** Variables associated with COVID-19 Severity

	**COVID-19 Severity**	
*Predictors*	*Odds Ratios*	*CI*
Recent cytotoxic therapy	0.63	0.18 – 2.19
Recent targeted therapy	1.89	0.64 – 5.55
Recent immunotherapy	0.51	0.19 – 1.39
No systemic therapy, ever	0.91	0.29 – 2.87
Melanoma as the primary cancer (Yes)	0.83	0.40 – 1.71
Age, per decade		
Linear	0.74	0.46 – 1.20
Quadratic	1.24	1.12 – 1.37
Sex (Male vs Female)	1.80	0.96 – 3.36
Race (Non-Hispanic White vs Other)	1.14	0.50 – 2.60
Sunbelt region (yes vs no)	0.35	0.17 – 0.75
Smoking status (Ever vs Never)	1.42	0.78 – 2.60
Obesity (yes vs no)	0.50	0.27 – 0.93
Cardiovascular comorbidities (yes vs no)	0.96	0.46 – 2.00
Renal disease (yes vs no)	1.81	0.72 – 4.56
Pulmonary comorbidities (yes vs no)	2.27	1.12 – 4.62
Diabetes (yes vs no)	2.12	1.01 – 4.47
Immunosuppressed (yes vs no)	1.55	0.66 – 3.64
ECOG status (1 vs 0)	0.93	0.42 – 2.08
ECOG status (2 + vs 0)	12.53	4.22 – 37.19
ECOG status (Unknown vs 0)	1.49	0.72 – 3.07
Cancer status (Active, stable/responding vs remission)	1.37	0.56 – 3.38
Cancer status (Active, progressing vs remission)	3.22	1.21 – 8.58
Cancer status (Unknown vs remission)	1.05	0.38 – 2.91
COVID Diagnosis (May—Aug 2020 vs Jan—Apr 2020)	0.18	0.08 – 0.40
COVID Diagnosis (Sep—Dec 2020 vs Jan—Apr 2020)	0.17	0.08 – 0.38
COVID Diagnosis (Jan—Apr 2021 vs Jan—Apr 2020)	0.10	0.03 – 0.31
COVID Diagnosis (May—Aug 2021 vs Jan—Apr 2020)	0.18	0.05 – 0.63
COVID Diagnosis (Sep—Dec 2021 vs Jan—Apr 2020)	0.14	0.02 – 0.83

Because melanoma therapies can cause end-organ toxicities (e.g. immune-related adverse events impacting many different organs, and BRAF/MEK inhibitors causing cardiomyopathy), we assessed organ function in relation to systemic therapy. Among hospitalized patients, there was no obvious difference in brain natriuretic peptide, troponin, supplemental oxygen requirement, or C-reactive protein (Supplementary Table 2). This was true regardless of therapy class, although numbers were small and limited definitive conclusions.

Finally, we assessed 30- and 90-day mortality among patients with a history of melanoma (but no active cancer) and compared these with cancer survivors with other cancers, hypothesizing that patients with a history of melanoma may have fewer comorbid conditions and perhaps have other less quantifiable advantages (e.g. excess sun exposure correlating with a more active lifestyle). We observed approximately similar mortality at 30 and 90 days (15% vs. 12%; 17% vs. 16%) among patients with a history of melanoma compared with other cancers (Supplemental Table 3). On multivariable analyses, patients with melanoma had similar odds of 30-day mortality (OR 1.38, 95% CI 0.69 – 2.76) and 90-day mortality (OR 1.21, 95% CI 0.62 – 2.35). Factors associated with inferior survival are listed in Table 3, but broadly overlapped with factors associated with higher COVID-19 severity in the full melanoma cohort.

**Table 3:** Factors associated with 30 and 90 day mortality in patients without cancer or cancer treatment in the previous 3 months

	30 day mortality		90 day mortality	
	OR	CI	OR	CI
Cancer type (Melanoma vs Other)	1.38	0.69—2.76	1.21	0.62—2.35
Steroids as a COVID-19 treatment, ever	2.03	1.5—2.73	2.38	1.81—3.14
Age, per decade				
Linear	1.42	0.88—2.3	1.33	0.9—1.97
Quadratic	1.06	0.98—1.15	1.06	0.99—1.13
Sex (Male vs Female)	1.47	1.11—1.94	1.58	1.22—2.04
Race (Non-Hispanic White vs Other)	0.91	0.68—1.22	0.98	0.75—1.28
Sunbelt region (yes vs no)	0.70	0.47—1.06	0.78	0.54—1.12
Smoking status (Ever vs Never)	1.21	0.9—1.62	1.07	0.81—1.4
Obesity (yes vs no)	1.09	0.81—1.46	0.98	0.75—1.28
Cardiovascular comorbidities (yes vs no)	0.97	0.72—1.3	1.11	0.85—1.46
Renal disease (yes vs no)	1.35	1—1.82	1.41	1.06—1.86
Pulmonary comorbidities (yes vs no)	1.35	0.99—1.83	1.37	1.03—1.83
Diabetes (yes vs no)	1.30	0.98—1.73	1.16	0.89—1.51
Immunosuppressed (yes vs no)	1.37	0.84—2.25	1.70	1.1—2.63
ECOG status (1 vs 0)	1.86	1.18—2.95	1.94	1.29—2.93
ECOG status (2 + vs 0)	3.44	2.13—5.55	4.00	2.58—6.18
ECOG status (Unknown vs 0)	1.66	1.11—2.47	1.52	1.06—2.17
COVID Diagnosis (2021 vs 2020)	0.43	0.29—0.65	0.42	0.29—0.61

## Discussion

In this study, we found that recent anti-cancer therapy, irrespective of modality, was not strongly associated with COVID-19 severity among patients with melanoma. Further, recent therapy did not obviously predispose patients to compromised organ function, a finding of clinical relevance given the varied and frequent organ involvement with immune-related adverse events. To our knowledge, this is the first systematic effort to study outcomes following BRAF and MEK inhibitors and COVID-19. Finally, melanoma was associated with modestly inferior COVID-19 survival in univariate but not multivariable analyses in a cohort of cancer survivors, contrasting with our hypothesis that it might be associated with improved outcomes.

Several studies have demonstrated the impact of various cancer treatments on COVID-19 outcomes in patients with cancer. Certain treatments have been associated with inferior outcomes, including several multiagent chemotherapy regimens (rituximab + cyclophosphamide, doxorobucin, vincristine, and predisione [R-CHOP], platinum + etoposide) and B cell malignancy directed therapies [3, 6]. Initially, clinicians had a high degree of concern that immune checkpoint inhibitors might synergize with SARS-CoV2 to produce enhanced lung or other organ inflammation, based on theoretical concern and early reports [7, 8]. Subsequent studies however have largely suggested that these agents are not associated with inferior COVID-19 outcomes [3, 9]. Although subclinical effects are difficult to rule out, our study provides additional evidence that this type of relationship is not present, at least when assessing COVID-19 severity and measures of organ function in hospitalized patients. In addition, our study is the largest to specifically study melanoma, where high rates of combination ipilimumab and nivolumab are used. This combination is associated with pneumonitis, myocarditis, and fatal toxicities more often than single agent anti-PD-1 regimens [10–12].

Similarly, we assessed targeted therapy in melanoma, specifically BRAF and MEK inhibitors. These agents are associated with modest rates of myocardial dysfunction, pyrexia, and elevated liver transaminase levels, all of which may confound or delay a diagnosis of COVID-19 infection, or could worsen organ function in the setting of concomitant COVID-19 infection. Although there was a potential trend towards increasing COVID-19 severity in this cohort, it was far from statistically significant, and there was no evidence of increasing organ dysfunction. Thus, BRAF and MEK inhibitors appear to join other non-chemotherapy agents that have no obvious impact on COVID-19 outcomes [3, 13].

Patients with a history of melanoma (defined as those without active melanoma or melanoma therapy in the prior 3 months) had a marginally inferior survival on univariate analyses compared with survivors of other cancers, although this difference was not present after adjustment for other prognostic variables. We originally hypothesized that melanoma may be associated with fewer co-morbidities than many other cancers since it is not associated with smoking, and thus more active lifestyle and better COVID-19 outcomes [14]. It is possible though that patients with melanoma do not have less co-morbidities, or that adjustments for comorbidities masked this difference. Regardless, despite the large number of patients, there were only 89 melanoma survivors, limiting the generalizability of these conclusions.

This study has limitations: even though it came from the largest registry of patients with cancer and COVID-19 (CCC19), the number of patients receiving different therapies remained modest. Further, teasing out other potentially relevant interactions (e.g. whether a patient was early on therapy, or whether only patients of a certain age on various therapies were impacted) was particularly difficult. However, we do provide evidence for overall safety of these regimens in patients with advanced melanoma.

In conclusion, patients with melanoma on targeted or immune therapy did not experience more severe COVID-19 or obviously enhanced organ damage in this study. Patients with a history of melanoma paradoxically appeared to have worse clinical outcomes compared with unselected cancer survivors, although this difference was not significant on multivariable modeling. This study adds to the growing body of evidence for the safety of most immune and targeted therapies in the context of COVID-19, and suggests additional studies into risk factors for adverse outcomes in cancer patients and survivors.

## Supplementary Information

**Additional file 1:** **Supplemental Table 1.** Outcomes summarized over full patient cohort. **Supplementary Table 2.** Organ function in hospitalized patients based on therapy type. **Supplemental Table 3.** Survival in patients without active cancer or treatment in the previous 3 months.

## Data Availability

The data that support the findings of this study are available from COVID-19 and Cancer Consortium (CCC-19) but restrictions apply to the availability of these data, which were used under license for the current study, and so are not publicly available. The dataset analyzed for the primary and secondary hypotheses will be made available upon request; requests should be sent to contact@ccc19.org. Individual deidentified patient data with site identifiers removed and geographic region of patient residence masked to a level no smaller than U.S. Census Divisions will be made available to researchers who provide a methodologically sound proposal, and whose proposed use of the data has been approved by an independent review committee identified for this purpose. External proposals can be submitted beginning 6 months and up to 72 months after publication of this article; the CCC19 is open to additional collaborators as well. All proposals should be directed to contact@ccc19.org; to gain access, data requestors will need to sign a data access agreement.
