# Supplementary material for: Impact of COVID-19 in patients on active melanoma therapy and with history of melanoma
Source: BMC Cancer. 2023 Mar 23;23:265. doi: 10.1186/s12885-023-10708-6 (PMC10033295; doi:10.1186/s12885-023-10708-6)
Supplement: Supplementary file 1 — Additional file 1: Supplemental Table 1. Outcomes summarized over full patient cohort. Supplementary Table 2. Organ function in hospitalized patients based on therapy type. Supplemental Table 3. Survival in patients without active cancer or treatment in the previous 3 months. [file 12885_2023_10708_MOESM1_ESM.docx]

Supplemental Table 1: Outcomes summarized over full patient cohort

| Outcomes | All patients |
| --- | --- |
|  | N = 307 |
| Hospitalization | 127 (41%) |
| No supplemental O2 | 42 (14%) |
| Supplemental O2 | 85 (28%) |
| ICU admission | 40 (13%) |
| Received mechanical ventilation | 20 (7%) |
| Death at any time* | 44 (14%) |
| Follow up time, days [Median (IQR)] | 90 (30-180) |

*Deaths were attributed to COVID-19: 24 (55%), cancer: 6 (14%), both COVID-19 and cancer: 3 (7%), other (this could include causes that were related to COVID-19, such as cerebral hemorrhage): 6 (14%), Unknown: 5 (11%)

Supplementary Table 2: Organ function in hospitalized patients based on therapy type.

| Outcomes | Recent cytotoxic therapy | Recent targeted therapy | Recent immunotherapy | No recent systemic therapy |
| --- | --- | --- | --- | --- |
|  | N = 9 | N = 15 | N = 18 | N = 81 |
| BNP |  |  |  |  |
| Normal | 0 (0%) | 0 (0%) | 2 (11%) | 14 (17%) |
| Abnormal | 1 (11%) | 1 (7%) | 4 (22%) | 12 (15%) |
| Missing/Unknown | 8 (89%) | 14 (93%) | 12 (67%) | 55 (68%) |
| CRP |  |  |  |  |
| Normal | 0 (0%) | 0 (0%) | 0 (0%) | 3 (4%) |
| Abnormal | 1 (11%) | 5 (33%) | 11 (61%) | 40 (49%) |
| Missing/Unknown | 8 (89%) | 10 (67%) | 7 (39%) | 38 (47%) |
| Troponin |  |  |  |  |
| Normal | 0 (0%) | 3 (20%) | 7 (39%) | 32 (40%) |
| Abnormal | 0 (0%) | 2 (13%) | 3 (17%) | 20 (25%) |
| Missing/Unknown | 9 (100%) | 10 (67%) | 8 (44%) | 29 (36%) |
| Supplemental O2 |  |  |  |  |
| Yes | 4 (44%) | 4 (27%) | 13 (72%) | 63 (78%) |
| No | 5 (56%) | 11 (73%) | 5 (28%) | 18 (22%) |

Supplemental Table 3 - Survival in patients without active cancer or treatment in the previous 3 months.

| Outcomes | All patients | Melanoma | Other cancer |
| --- | --- | --- | --- |
|  | N = 2381 | N = 89 | N = 2292 |
| 30-day mortality | 299 (13%) | 13 (15%) | 286 (12%) |
| 90-day mortality | 376 (16%) | 15 (17%) | 361 (16%) |
